# *Lactococcus lactis* subsp. *cremoris* YRC3780 modifies function of mesenteric lymph node dendritic cells to modulate the balance of T cell differentiation inducing regulatory T cells

**DOI:** 10.3389/fimmu.2024.1395380

**Published:** 2024-07-08

**Authors:** Ryogo Nakagawa, Wenting Gu, Hibine Mizobuchi, Shuhei Kodera, Tomohiro Takano, Yimei Wang, Ikumi Fujioka, Kenji Uchida, Haruyo Nakajima-Adachi, Satoshi Hachimura

**Affiliations:** ^1^ Research Center for Food Safety, Graduate School of Agricultural and Life Sciences, The University of Tokyo, Tokyo, Japan; ^2^ R&D Center, Yotsuba Milk Products, Co., Ltd., Kitahiroshima, Japan

**Keywords:** dendritic cells, regulatory T cells, intestine, lactic acid bacteria, allergy

## Abstract

**Introduction:**

The intestinal immune system plays a pivotal role in the induction of immune responses against food. In the case of T cell response, dendritic cells (DCs) are especially important. However, the regulation of immune responses to food by intestinal DCs has been poorly described. In this study, we analyzed the effect of *Lactococcus lactis* subsp. *cremoris* YRC3780, a lactic acid bacterial strain isolated from kefir, a traditional fermented milk product, on the immune responses induced by antigen presentation by intestinal DCs to T cells as well as the mechanism of action of these immunomodulatory effects. It has been shown that *L. cremoris* YRC3780 ameliorates the symptoms of pollinosis in both animal and human studies.

**Methods:**

CD11c^+^ cells from mesenteric lymph nodes (MLNs) of BALB/c mice were cultured as MLN DCs with *L. cremoris* YRC3780 and expression of genes inducing regulatory T cells (Tregs) was examined by qPCR. In addition, MLN DCs were cocultured with CD4^+^ T cells from DO11.10 transgenic mice expressing an ovalbumin (OVA)-specific TCR and the OVA antigen peptide and *L. cremoris* YRC3780. Induction of Tregs was examined by flow cytometry, gene expression was analyzed by DNA microarray and qPCR, and the production of cytokines was measured by ELISA. MLN DCs from TLR2-deficient mice and components of *L. cremoris* YRC3780 were used to examine the recognition of YRC3780 by MLN DCs.

**Results:**

*L. cremoris* YRC3780 enhanced the expression of genes involved in Treg induction in MLN DCs and induced Foxp3^+^CD4^+^T cells in an MLN DC and CD4^+^ T-cell co-culture system. The effect on MLN DCs was likely mediated by receptors other than TLR2. Together with microarray analyses of CD4^+^ T cell gene expression and cytokine ELISA, it was demonstrated that *L. cremoris* YRC3780 promoted the induction of Th1 and Tregs, and regulated the balance of Th1/Th2 and Treg/Th17 cells involving multiple genes via the antigen-presentation of MLN DCs.

**Discussion:**

Our findings provide insights into the modulation of intestinal immune responses mediated by DCs and the antiallergic effects of lactic acid bacteria.

## 1 Introduction

The intestinal immune system plays a pivotal role in the induction of immune responses against food. In the case of the T cell response, dendritic cells (DCs) are important and, intestinal DCs have a greater ability to induce regulatory T cells (Tregs) than do DCs from other sites (1). Therefore, the immune response to food components is likely to be affected by the characteristics of intestinal DCs. There are multiple reports on the effects of food components on intestinal DCs (1–10); however, the regulation of immune responses to food by intestinal DCs has not been fully elucidated. Concerning the mechanisms underlying DC-mediated enhancement of Treg induction, one study showed that a *Lactobacillus* strain enhanced the retinal dehydrogenase (RALDH) activity of mesenteric lymph node (MLN) DCs (3) and another study reported that two probiotic *Lactobacillus* strains induced integrin αvβ8 expression (4), but such regulation needs to be examined further. In the present study, we examined the effect of *Lactococcus lactis* subsp. *cremoris* YRC3780, a lactic acid bacterial strain isolated from kefir, a traditional fermented milk product of the Caucasus region, on the responses mediated by murine MLN DCs.

It has been shown that *L. cremoris* YRC3780 ameliorates the symptoms of pollinosis in both animal and human studies (11, 12). In an atopic dermatitis-like murine model of skin inflammation, oral administration of *L. cremoris* YRC3780 has alleviated allergen-induced dermal responses (13). It was demonstrated in this previous study that *L. cremoris* YRC3780 enhanced IFN-γ and IL-12 production and inhibited IL-4 production by allergen-specific CD4^+^ T cells in the presence of various types of immune cells including intestinal DCs (13).

In the present study, we examined in further detail the effect of *L. cremoris* YRC3780 on intestinal immune responses mediated by intestinal DCs. The gene expression of MLN DCs, and the CD4^+^ T cell responses induced by these MLN DCs were elucidated with particular attention to the induction of Tregs. The use of MLN DCs enabled us to show that this lactic acid bacterium promoted the induction of T helper (Th) 1 cells and Tregs, and suppressed the induction of Th2 cells, thus regulating the balances of Th1/Th2 and Treg/Th17 cells, via antigen presentation by intestinal DCs.

## 2 Materials and methods

### 2.1 Mice

BALB/c mice were purchased from CLEA Japan (Shizuoka, Japan) or Charles River Laboratories Japan (Yokohama, Japan). DO11.10 mice (14) and RAG2-deficient/DO11.10 mice (DO11.10 mice deficient in recombination-activating gene 2) on a BALB/c background were maintained and obtained from Sankyo Labo Service (Tokyo, Japan). Toll-like receptor (TLR)2-deficient mice (15) on a BALB/c background were purchased from Oriental Bioservice (Kyoto, Japan). The mice were fed CE-2 diet (CLEA Japan) and distilled deionized water ad libitum and maintained in a specific-pathogen-free environment in a temperature-controlled room, under a 12-h light-dark cycle. Female BALB/c mice 6 to 10 weeks old, female DO11.10 mice and RAG2-deficient/DO11.10 mice 10 to 22 weeks old, and male TLR2-deficient mice and their control BALB/c mice 9 to 10 weeks old were used. All experiments were performed in accordance with the Guidelines for the Care and Use of Laboratory Animals of the University of Tokyo and were approved by the Experimental Animal Ethics Committee of the Graduate School of Agricultural and Life Sciences, the University of Tokyo (approval numbers P19-025, P21-044, and P22-056).

### 2.2 Lactic acid bacteria

*L. cremoris* YRC3780 was inoculated into M17 broth and cultured for 16 h at 30°C. The bacterial cells were then harvested by centrifugation at 8000 rpm for 10 min at 4°C, washed twice, and resuspended in sterilized distilled water. Following heat treatment for 10 min at 100°C, the cells were lyophilized (3). *L. cremoris* JCM 16167 was prepared by using the same procedures except that the heat treatment was at 110°C for 10 min. In the experiment using *L. cremoris* JCM 16167, heat treatment of *L. cremoris* YRC3780 was also performed at 110°C for 10 min.

In addition, the *L. cremoris* YRC3780 bacterial cells were fractionated as follows. The bacterial cells were sonicated, heat-treated for 10 min at 100°C and centrifuged at 1000 g for 5 min. The supernatant was then centrifuged at 13000 g for 20 min. The supernatant was used as the cytoplasmic fraction. The precipitate was treated with sodium dodecyl sulfate (SDS) for 30 min, enzymatically treated with trypsin for 3 h at 37°C and centrifuged at 13000 g for 20 min. The precipitate was treated with SDS for 30 min, washed and then treated with 3% trichloroacetic acid for 24 h at 4°C and for 24 h at 37°C to obtain the cell-wall fraction. Moreover, to obtain the RNA fraction, RNA in the bacterial cells was extracted using an RNeasy Mini Kit (QIAGEN, Hilden, Germany) according to the protocol provided by the manufacturer. To obtain the DNA fraction, DNA in the bacterial cells was extracted using a DNase Blood & Tissue Kit (QIAGEN) according to the protocol provided.

### 2.3 Media and reagents

RPMI 1640 (Nissui Pharmaceutical, Tokyo, Japan) containing 100 U/mL penicillin G potassium (Meiji Seika Pharma, Tokyo, Japan), 100 μg/mL streptomycin sulfate (Meiji Seika Pharma), 50 μM 2-mercaptoethanol (Tokyo Chemical Industry, Tokyo, Japan), 0.03% l-glutamine (FUJIFILM Wako Pure Chemical Corporation, Osaka, Japan) and 0.2% sodium hydrogen carbonate (FUJIFILM Wako Pure Chemical Corporation) was prepared with heat-inactivated fetal calf serum (FCS) (Gibco, Carlsbad, CA, United States; Sigma Aldrich, St Louis, United States) supplemented to a final concentration of 10% and used as 10% FCS-RPMI.

For cell culture, recombinant human transforming growth factor (TGF)-β1 and recombinant human latent TGF-β1 were purchased from R&D Systems (Minneapolis, MN, United States); DMSO was purchased from Sigma Aldrich (St Louis, MO, United States); and OVA323-339 peptide (OVAp) (ISQAVHAAHAEINEAGR) was purchased from Eurofins Genomics (Tokyo, Japan).

For cytokine analysis by enzyme-linked immunosorbent assay (ELISA), purified rat anti-mouse IL-4 (11B11) and purified rat anti-mouse IFN-γ (R4-6A2) were used as primary antibodies for coating and were purchased from BD Biosciences (Franklin Lakes, NJ, United States). Biotin rat anti-mouse IL-4 (BVD6-24G2) and biotin rat anti-mouse IFN-γ (XMG1.2) were used as secondary antibodies and were also purchased from BD Biosciences. Recombinant IL-4 and recombinant IFN-γ for standard solutions were purchased from Peprotech (Cranbury, New Jersey, United States). Streptavidin-alkaline phosphatase was purchased from BD Biosciences. Disodium *p*-nitrophenylphosphate was purchased from Tokyo Chemical Industry as a chromogenic substrate for detection. Mouse TGF-β1 DuoSet ELISA kit and Reagent Diluent Concentrate 1 were purchased from R&D Systems and the TMB Substrate Reagent Set was purchased from BD Biosciences for the measurement of TGF-β1.

For flow cytometry, purified anti-mouse CD16/32 antibody (93), APC/Cy7 anti-mouse CD11c antibody (N418), FITC anti-mouse TCR DO11.10 antibody (KJ1-26), APC anti-mouse CD4 antibody (GK1.5) and PE rat IgG2b, κ isotype control antibody (RTK4530) were purchased from BioLegend (San Diego, CA, United States); FOXP3 monoclonal antibody PE (FJK-16 s), FOXP3 monoclonal antibody PE/Cy7 (FJK-16 s) and IL-10 monoclonal antibody PE (JES5-16E3) were purchased from Thermo Fisher Scientific (Waltham, MA, United States).

### 2.4 Immune cell preparation

For DC isolation, MLNs obtained from BALB/c mice or TLR2-deficient mice were digested in 10% FCS-RPMI containing 0.5 mg/mL collagenase (FUJIFILM Wako Pure Chemical Corporation) with 10 μg/mL DNase I (Roche Diagnostics, Mannheim, Germany), and a cell suspension was obtained by filtering the digestate. CD11c^+^ cells were magnetically purified from the obtained whole cells, using the magnetic activated cell sorting (MACS) cell separation system (Miltenyi Biotec, Bergisch Gladbach, Germany) according to the manufacturer’s instructions. To obtain high-purity CD11c^+^ cells as DCs for culture, CD11c^+^ purification with MACS was conducted twice.

For CD4^+^-T cell isolation, splenocytes (SPLs) were obtained from DO11.10 mice, RAG2-deficient/DO11.10 mice, TLR2-deficient mice, and control mice. CD4^+^ cells from whole SPL were separated using the MACS system. Cells were isolated from multiple mice and pooled to obtain the required numbers of cells.

For analysis of the DNA microarray, MACS-enriched SPL CD4^+^T cells were sorted by using a FACS Aria (BD Biosciences) to obtain CD4^+^Kj1.26^+^ T cells. PBS containing 2% FCS was used as the staining and washing buffer.

### 2.5 Cell culture

In the MLN DC monoculture system, MLN DCs (1 × 10^5^ cells for qRT-PCR; 2-4 × 10^5^ cells for flow cytometry) from BALB/c mice or TLR2-deficient mice were cultured with *L. cremoris* YRC3780 (100 µg/mL unless otherwise indicated) or each fraction of *L. cremoris* YRC3780 (10 or 100 µg/mL). Cultures were also conducted in the presence of latent TGF-β1 (10 ng/mL). The cells were cultured for 18 h for qRT-PCR and 48 h for ELISA and flow cytometry in a 96-well flat-bottomed plate (Corning, New York, NY, United States) in 200 μL of 10% FCS-RPMI under a 5% CO2 humidified atmosphere at 37°C.

In the MLN DC and T cell coculture system, MLN DCs (2 × 10^4^ cells) from BALB/c mice and SPL CD4^+^T cells (2 × 10^5^ cells) from DO11.10 or RAG2-deficient/DO11.10 mice were cocultured with *L. cremoris* YRC3780 in the presence of OVAp (10 nM). Cultures were also conducted in the presence of latent TGF-β1 (10 ng/mL). For analysis of intracellular IL-10, MLN DCs (4 × 10^4^ cells) from BALB/c mice and SPL CD4^+^T cells (4 × 10^5^ cells) from RAG2-deficient/DO11.10 mice were cocultured with *L. cremoris* YRC3780 in the presence of OVAp (100 nM), and GolgiStop protein transport inhibitor (0.1%; BD Biosciences) was added in the last 4 h. Cultures were conducted for 48 h or 60 h for qRT-PCR, 60 h for DNA microarray, 48 h for ELISA and 72 h for flow cytometry as in the case of MLN DCs mono-culture system.

### 2.6 qRT-PCR

Total RNA was extracted from the cells by using QIAShredder (QIAGEN, Hilden, Germany) and an RNeasy Mini Kit (QIAGEN). cDNA was synthesized with SuperScript VILO MasterMix (Thermo Fisher Scientific). Subsequently, real-time PCR was performed with the QuantiTect SYBR Green PCR Kit (QIAGEN) and LightCycler (Roche Diagnostics) or a CFX Connect Real-Time PCR Detection System (Bio-Rad, CA, United States). cDNA was amplified with specific primers (**Supplementary Table**). Relative gene expression was calculated by assuming that the targeted cDNA was doubled at one cycle. The results were normalized to GAPDH gene expression as the internal control.

### 2.7 DNA microarray

The analyses were entrusted to Cell Innovator (Fukuoka, Japan). The extracted total RNA was analyzed by using SurePrint G3 Mouse GE 8x60K Ver. 2.0 (Agilent Technologies, Santa Clara, CA, United States) as follows. Labeling was performed by using a Low Input Quick Amp Labeling Kit (Agilent Technologies). Then, hybridization, washing and scanning were performed according to the manufacturer’s instructions. The data were quantified by using Feature Extraction software (Agilent Technologies) and normalized by the quantile method using the statistical analysis software R.

### 2.8 ELISA

Cytokine production in the culture supernatants was measured by using specific sandwich ELISA. IL-4 and IFN-γ were quantified as follows. Ninety-six-well immunoplates (Nunc, Roskilde, Denmark) were coated with primary antibodies and incubated overnight at 4°C. After the plates had been washed and blocked for 2 h at room temperature, the samples and standards were added and incubated for 2 h at room temperature or overnight at 4°C. Bound cytokines were detected with secondary antibodies, and incubated for 1 h at room temperature. The plates were then washed and the enzyme was added and incubated for 30 min at room temperature. After washing, the chromogenic substrate was added. Absorbance was determined at a wavelength of 405 nm. TGF-β was quantified by a Mouse TGF-β1 DuoSet ELISA kit (R&D Systems) according to the manufacturer’s instructions. For activation of latent TGF-β, samples were reacted with 1 M hydrochloric acid (FUJIFILM Wako Pure Chemical Corporation) for 10 min at room temperature, then neutralized by adding 1.2 M sodium hydroxide ((FUJIFILM Wako Pure Chemical Corporation)/0.5 M HEPES ((FUJIFILM Wako Pure Chemical Corporation) solution. Absorbance was measured with a Microplate Reader Model 680 (Bio-Rad) and data were analyzed with Microplate Manager III (Bio-Rad).

### 2.9 Flow cytometry

Cells were stained as follows. Anti-CD16/32 antibody was added for 15 min at 4°C to block the Fc receptor. After the cells had been washed, target proteins on the cell surfaces were stained with fluorescently labeled antibodies for 20 min at 4°C. To stain dead cells, propidium iodide (Sigma Aldrich) was added to the samples after staining of the cell surfaces with antibodies, and the samples were then washed out immediately. For the intracellular staining described below, the fixable viability dye eFluor 780 (Thermo Fisher Scientific) was added before fixation, instead of propidium iodide. Intracellular Foxp3 was stained by using a Foxp3/Transcription Factor Staining Buffer Set (Thermo Fisher Scientific) according to the manufacturer’s instructions. Intracellular IL-10 was also stained according to the same instructions in this staining kit: After cell surface staining, the cells were fixed and permeabilized for 2 h at 4°C with the working solution in the staining kit. Anti-CD16/32 antibody was then added for 15 min at 4°C to block the Fc receptor, and the IL-10 was then stained intracellularly with fluorescently labeled IL-10 monoclonal antibody for 30 min at 4°C.

An ALDEFLUOR assay kit (StemCell Technologies, Vancouver, Canada) was used to evaluate RALDH activity. After we had performed cell surface staining and dead cell staining with propidium iodide as described above, ALDEFLUOR reagent containing BAAA (BODIPY-aminoacetaldehyde) was added and incubated for 15 min at 37°C. When BAAA reacts with aldehyde dehydrogenase (ALDH), it produces a green fluorescent compound BAA (BODIPY-aminoacetate), which is retained inside the cell. RALDH activity was confirmed by comparison with a control sample containing an ALDH inhibitor (N,N-diethylaminobenzaldehyde; DEAB).

The fluorescence levels were measured by FACS Verse (BD Biosciences) and data were analyzed using FLOWJO software (BD Biosciences).

### 2.10 Statistical analysis

The results are shown as the mean ± SD. Student’s *t*-test or Tukey’s HSD test was used for statistical analyses. The R package limma was used for DNA microarray analyses, and two samples from each of the two experiments were combined (n=4). Differences were considered significant at *p* < 0.05.

## 3 Results

### 3.1 *L. cremoris* YRC3780 enhances the gene expression of Treg-inducing factors in MLN DC cultures

**Figure 1:**
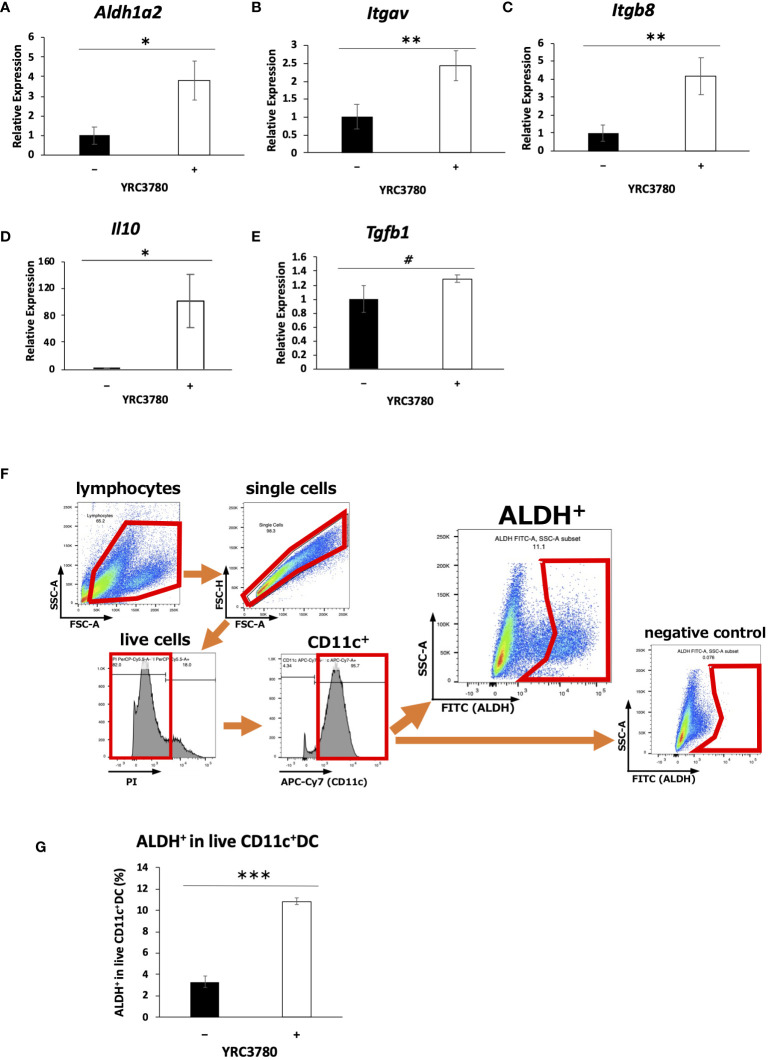
*L. cremoris* YRC3780 enhanced gene expression of Treg-inducing factors in MLN DC cultures. MLN DCs from BALB/c mice were cultured in the presence (100 μg/mL) or absence of *L. cremoris* YRC3780. Expression of *Aldh1a2* **(A)**, *Itgav* **(B)**, *Itgb8* **(C)**, *Il10* **(D)** and *Tgfb1* **(E)** was measured by qRT-PCR. The percentage of ALDH^+^DCs among live CD11c^+^DCs was analyzed by flow cytometry **(F, G)**. The plot shows one representative dataset from two independent experiments. Student’s *t*-test was used for statistical analysis (#*p*<0.1, *p <0.05, **p<0.01, ***p<0.001).

CD11c^+^DCs derived from MLN of BALB/c mice were cultured in the presence of *L. cremoris* YRC3780 (**Figure 1**). Expression of *Aldh1a2* (encoding RALDH2), *Itgav* (integrin αv), and *Itgb8* (integrin β8) and *Il10* (IL-10) was significantly up-regulated by the addition of *L. cremoris* YRC3780 (**Figures 1A–D**). Gene expression of TGF-β was also analyzed, although not significant, a numerical increase was observed (**Figure 1E**). We also found that such enhancement of gene expression in DC was observable in another *L. cremoris* type strain JCM 16167 albeit at levels a little lower than that of YRC 3780 (**Supplementary Figure S1**). Because the gene expression of RALDH2, an enzyme involved in the production of retinoic acid, was enhanced, we analyzed the percentage of ALDH^+^DCs among live CD11c^+^DCs by flow cytometry under the same culture conditions as above to determine whether the enzyme activity was actually enhanced. The percentage of ALDH^+^DCs with RALDH enzyme activity was significantly increased by the addition of *L. cremoris* YRC3780 (**Figures 1F, G**). Integrins αv and β8 are cell adhesion molecules that activate TGF-β. As the expression of the genes encoding these integrins was enhanced, we added inactive TGF-β (latent TGF-β) under the same culture conditions as above in order to confirm whether latent TGF-β was actually converted to the active TGF-β. The amounts of cytokines in the supernatants were analyzed by ELISA, but neither the amount of latent TGF-β nor active TGF-β was significantly changed by the addition of *L. cremoris* YRC3780 (**Supplementary Figure S2**).

**Figure 2:**
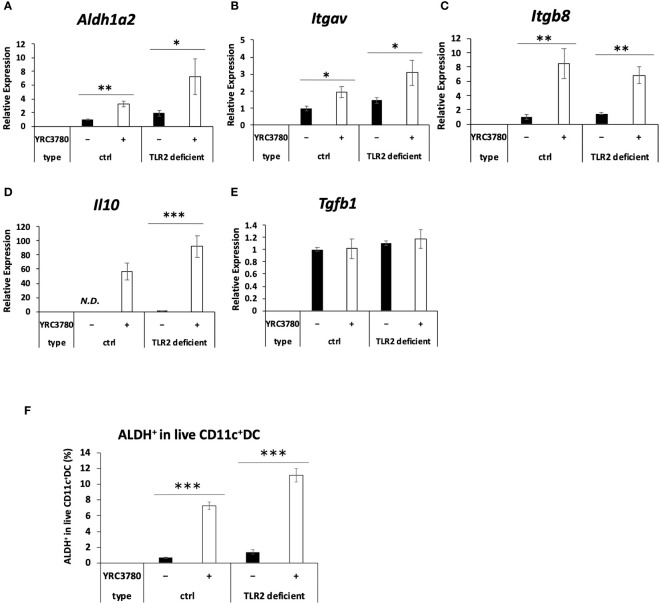
Effects of *L. cremoris* YRC3780 on the gene expression of Treg-inducing factors in MLN DC cultures from TLR2-deficient mice. MLN DCs from TLR2-deficient or wild type BALB/c mice (ctrl) were cultured in the presence or absence of *L. cremoris* YRC3780. Expression of *Aldh1a2* **(A)**,*Itgav* **(B)**, *Itgb8* **(C)**, *Il10* **(D)** and *Tgfb1* **(E)** was measured by qRT-PCR. The percentage of ALDH^+^DCs among live CD11c^+^DCs was analyzed by flow cytometry **(F)**. The plot shows one representative dataset from two independent experiments. Student’s *t*-test was used for statistical analysis (*p <0.05, **p<0.01, ***p<0.001). ND, not detected.

**Figure 3:**
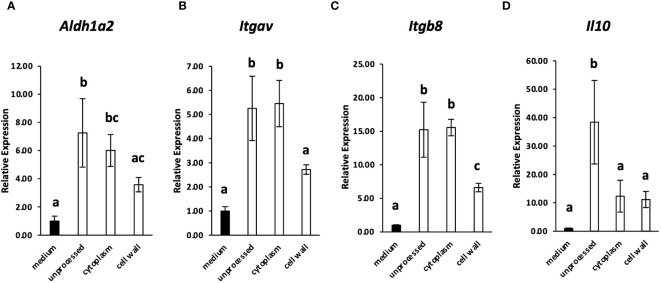
The effect of the cytoplasmic and cell-wall fraction of *L. cremoris* YRC3780 on the gene expression of Treg-inducing factors in MLN DC cultures. MLN DCs from BALB/c mice were cultured in medium only or in the presence (100 μg/mL) of unprocessed *L. cremoris* YRC3780, the cytoplasmic fraction, or the cell-wall fraction of YRC3780. Expression of *Aldh1a2* **(A)**, *Itgav* **(B)**, *Itgb8* **(C)** and *Il10* **(D)** was measured by qRT-PCR. The plot shows one representative dataset from two independent experiments. Tukey’s HSD test was used for statistical analysis. Values not sharing a common letter are significantly different.

Because many lactic acid bacteria strains are recognized by immune cells via TLR2 (2, 16), the involvement of TLR2 in the recognition of *L. cremoris* YRC3780 by MLN DCs was examined by using TLR2-deficient mice. CD11c^+^DCs from the MLN of TLR2-deficient mice or BALB/c mice as controls were cultured in the presence of *L. cremoris* YRC3780 (**Figure 2**). Expression of *Aldh1a2*, *Itgav*, *Itgb8* and *Il10* was significantly increased by the addition of *L. cremoris* YRC3780 in both control and TLR2-deficient mice, whereas the expression of *Tgfb1* did not change (**Figures 2A–E**). We also analyzed the percentage of ALDH^+^DCs among live CD11c^+^DCs by flow cytometry. Adding *L. cremoris* YRC3780 significantly increased the percentage of ALDH^+^DCs with RALDH enzyme activity in both control and TLR2-deficient mice (**Supplementary Figure S3**, **Figure 2F**). To elucidate the component(s) of *L. cremoris* YRC3780 that induced expression of the abovementioned genes, the cytoplasmic fraction and cell-wall fraction were prepared, and added to the DC cultures. Expression of the four genes stably upregulated by the unprocessed heat-treated *L. cremoris* YRC3780 preparation *Aldh1a2*, *Itgav*, *Itgb8* and *Il10* was then analyzed. The cytoplasmic fraction induced expression of *Aldh1a2, Itgav*, and *Itgb8*. The expression levels were comparable to that of unprocessed heat-treated YRC3780 (**Figure 3**). The cell-wall fraction significantly enhanced expression of *Itgb8* and tended to enhance expression of *Aldh1a2* in the sense that their expression levels were not significantly different compared to that induced by the cytoplasmic fraction (**Figure 3**). The results suggested that both components of cytoplasm and cell wall were recognized by the MLN DCs. We then further examined the activity of *L. cremoris* YRC3780 DNA and RNA that may have been contained in the cytoplasm fraction. RNA and DNA of *L. cremoris* YRC3780 significantly increased the expression of *Itgb8* (**Supplementary Figure S4**).

### 3.2 *L. cremoris* YRC3780 enhanced antigen-specific induction of Foxp3^+^CD4^+^T cells in MLN DC and T cell coculture system

**Figure 4:**
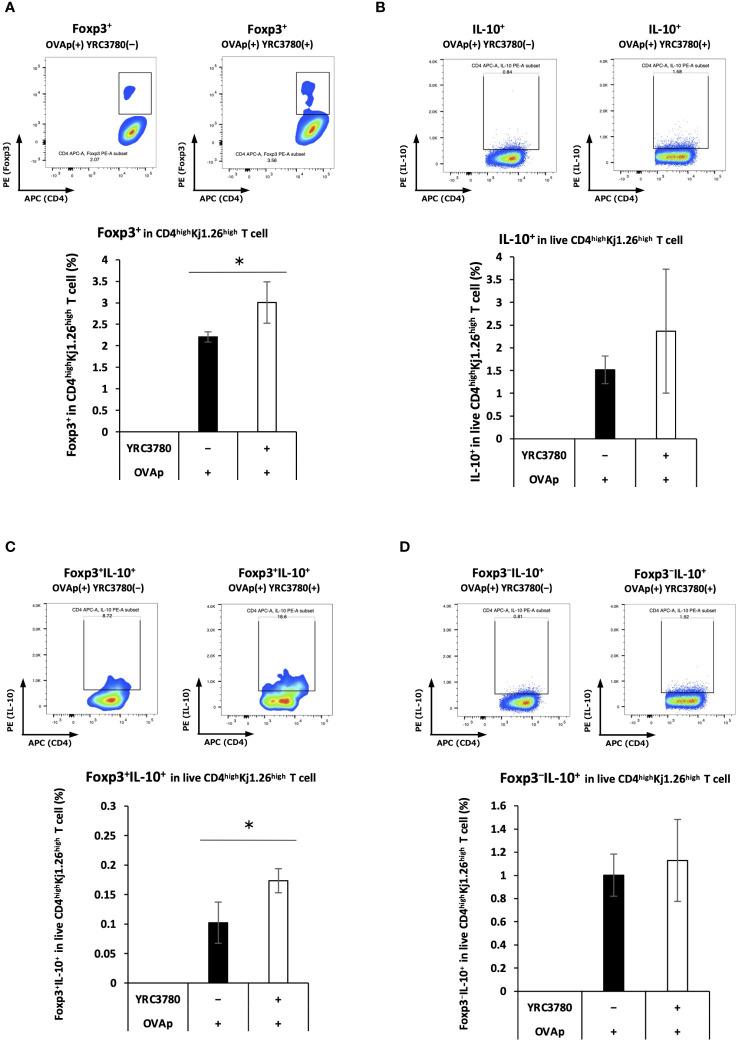
*L. cremoris* YRC3780 enhanced antigen-specific induction of Foxp3^+^CD4^+^T cells in the MLN DC and T cell coculture system. CD11c^+^DCs from the MLNs of BALB/c mice and CD4^+^T cells from the SPLs of DO11.10 or RAG2-deficient/DO11.10 mice were incubated with *L. cremoris* YRC3780 and OVAp. The percentages of Foxp3-expressing **(A)**, IL-10-expressing **(B)**, Foxp3^+^IL-10^+^- **(C)** and Foxp3^-^IL-10^+^ **(D)** cells in CD4^high^Kj1.26^high^T cells were analyzed by flow cytometry. Representative flow cytometry plots are shown for each population of both groups. Each plot shows one representative dataset of two independent experiments. Student’s *t*-test was used for statistical analysis (*p <0.05).

*L. cremoris* YRC3780 enhanced the expression of factors (i.e. RALDH2 and integrin αvβ8) that induce Foxp3^+^ Tregs. To confirm the induction of Foxp3^+^ T cells, CD11c^+^DCs from the MLNs of BALB/c mice and CD4^+^T cells from the SPLs of OVA-specific TCR transgenic DO11.10 mice were cultured with *L. cremoris* YRC3780 and OVAp. The percentage of Foxp3-expressing cells in CD4^high^Kj1.26^high^T cells was analyzed by flow cytometry (**Supplementary Figure S5**). The percentage of Foxp3^+^T cells in the coculture system with DO11.10 mice-derived T cells was significantly increased by the addition of *L. cremoris* YRC3780 (**Figure 4A**).

Gene-expression analysis revealed that adding *L. cremoris* YRC3780 increased IL-10 expression in MLN DCs. As the presence of IL-10 enhances the induction of IL-10-producing Tregs, we examined Foxp3/IL-10 expression in CD4^+^ T cells in this culture system. CD11c^+^DCs from the MLNs of BALB/c mice and CD4^+^T cells from the SPLs of RAG2-deficient/DO11.10 mice were cocultured in the presence of *L. cremoris* YRC3780 and OVAp. The percentage of Foxp3/IL-10-expressing cells among live CD4^high^Kj1.26^high^T cells was analyzed by flow cytometry (**Supplementary Figure S6**). The percentage of Foxp3^+^IL-10^+^ T cells increased (**Figure 4C**). There was no significant change in the percentage of total IL-10^+^T cells (**Figure 4B**) nor Foxp3^−^IL-10^+^T cells (**Figure 4D**).

### 3.3 Effects of *L. cremoris* YRC3780 on gene expression of CD4^+^T cells in an MLN DC and T cell coculture system

**Figure 5:**
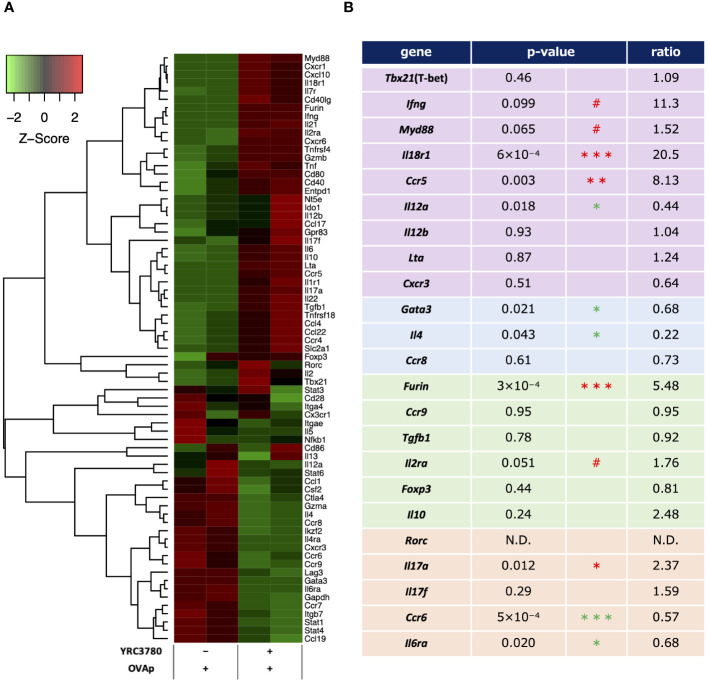
Microarray analyses of antigen-stimulated CD4^+^T cells in the presence or absence of *L. cremoris* YRC3780 in MLN DC and T cell coculture system. CD11c^+^DCs from BALB/c mouse MLNs and CD4^+^T cells from RAG2-deficient/DO11.10 mouse SPL were cocultured in the presence or absence of *L. cremoris* YRC3780 and OVAp. Antigen-specific (Kj1.26^+^) CD4^+^ T cells were purified by flow cytometry, and gene expression in these cells was analyzed by DNA microarray. Signals for genes associated with T-cell differentiation were standardized by Z-score normalization using the R package gene filter and represented as a heatmap by using the R package gplots **(A)**. The heatmap shows one representative data set of two independent experiments. The p-values and ratios of individual T subset-related genes were calculated from these two data sets **(B)**. The limma R package was used for statistical analysis (#*p*<0.1, *p <0.05, **p<0.01, *** *p*<0.001; increase in red, decrease in green).

To examine the effects of *L. cremoris* YRC3780 on T-cell differentiation by MLN DCs, CD11c^+^DCs from BALB/c mouse MLNs and CD4^+^T cells from RAG2-deficient/DO11.10 mouse SPLs were cocultured in the presence of *L. cremoris* YRC3780 and OVAp. Antigen-specific (Kj1.26^+^) CD4^+^ T cells were purified by flow cytometry, and gene expression in these CD4^+^T cells was analyzed by DNA microarray. We identified the following genes characteristic of each CD4^+^T cell subset, and we observed the differential expression of these genes upon the addition of *L. cremoris* YRC3780 (**Figures 5A, B**).

Among Th1-related genes, the expression of *Ifng* (IFN-γ), *Myd88* (MyD88), *Il18r1* (IL-18R) and *Ccr5* (CCR5) was increased or tended to increase, whereas that of *Il12a* (IL-12p35) decreased. Among Th2-related genes, the expression of *Gata3* (GATA-3) and *Il4* (IL-4) decreased. Among Treg-related genes, although the expression of *Foxp3* (Foxp3) did not change significantly according to the microarray analyses, that of *Furin* (furin) increased. Among Th17-related genes, the expression of *Il17a* (IL-17A) increased and that of *Ccr6* (CCR6) and *Il6ra* (IL-6R) decreased. The expression of Th1-related genes such as *Lta* (LT-α; TNF-β) and *Cxcr3* (CXCR3), Th2-related genes such as *Ccr8* (CCR8), Treg-related genes such as *Ccr9* (CCR9), *Tgfb1* (TGF-β), *Il10* (IL-10), and *Il2ra* (IL-2R; CD25), and Th17-related genes such as *Rorc* (RORgt) and *Il17f* (IL-17F) was also evaluated, but no significant changes were detected.

**Figure 6:**
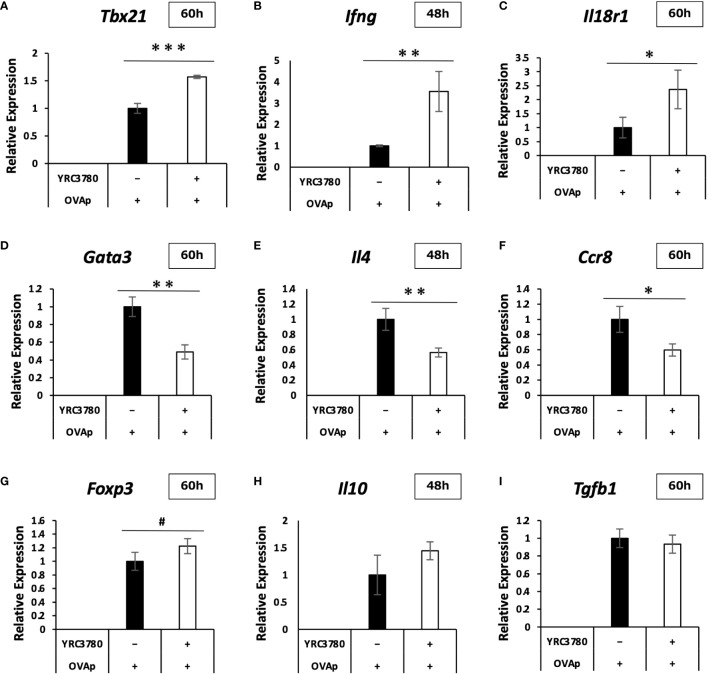
Effects of *L. cremoris* YRC3780 on gene expression of CD4^+^T cells in MLN DC and T cell coculture system. CD11c^+^DCs from BALB/c mouse MLNs and CD4^+^T cells from RAG2-deficient/DO11.10 mouse SPL were cocultured in the presence of *L. cremoris* YRC3780 and OVAp. qRT-PCR was used to analyze the expression levels of representative CD4^+-^T-cell subset-related genes; *Tbx21* **(A)**, *Ifng* **(B)**, *Il18r1* **(C)**, *Gata3* **(D)**, *Il4* **(E)**, *Ccr8* **(F)**, *Foxp3* **(G)**, *Il10* **(H)**, and *Tgfb1* **(I)**. The plot shows one representative dataset of two independent experiments. Student’s *t*-test was used for statistical analysis (#*p*<0.1, *p <0.05, **p<0.01, ***p<0.001).

Under the same culture conditions as above, qRT-PCR was used to analyze the expression levels of representative genes in the CD4^+^T cell subsets, as well as genes for which the expression varied, and important genes in the intestinal immune system. The results of the qRT-PCR analysis showed that the increases and decreases in the expression of each gene caused by the addition of *L. cremoris* YRC3780 were generally consistent with the results of the DNA microarray analysis (**Figures 6A–I**). In the case of some genes, expression that did not change significantly according to the microarray analyses was more clearly altered according to the qRT-PCR analyses. Specifically, the expression of *Tbx21*, *Ifng* and *Il18r1*, which are Th1 response-related genes, was significantly increased; that of *Gata3*, *Il4* and *Ccr8*, which are Th2 response-related genes, was significantly decreased, and that of *Foxp3*, which is a Treg-response-related gene, tended to increase. These results suggested that *L. cremoris* YRC3780 promoted the induction of Th1 and Tregs, while inhibiting the induction of Th2 cells, thus regulating the Th1-Th2 and Treg-Th17 balance.

### 3.4 Effects of *L. cremoris* YRC3780 on cytokine production by CD4^+^T cells in the MLN DC and T cell coculture system

**Figure 7:**
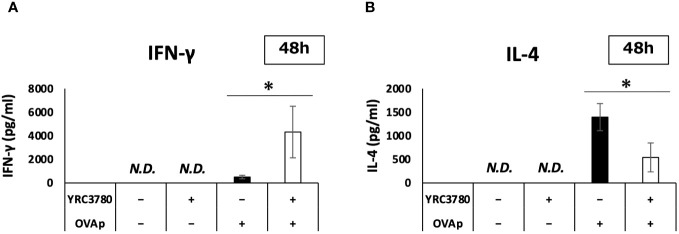
Effects of *L. cremoris* YRC3780 on cytokine production in MLN DC and T cell coculture system. CD11c^+^DCs from the MLNs of BALB/c mice and CD4^+^T cells from the SPL of RAG2-deficient/DO11.10 mice were cocultured in the presence or absence of *L. cremoris* YRC3780 and OVAp. The amounts of IFN-γ **(A)** and IL-4 **(B)** in the supernatants were analyzed by ELISA. The plot shows one representative dataset of two independent experiments. Student’s *t*-test was used for statistical analysis (*p <0.05). ND, not detected.

On the basis of the gene expression results in the MLN DC and T cell coculture system, we used ELISA to analyze cytokine production. We cocultured CD11c^+^DCs from the MLNs of BALB/c mice with CD4^+^T cells from the SPLs of RAG2-deficient/DO11.10 mice in the presence or absence of *L. cremoris* YRC3780 and OVAp. The amounts of cytokines in the supernatants were analyzed by ELISA. The results showed that adding *L. cremoris* YRC3780 significantly increased the production of IFN-γ and significantly decreased that of IL-4 (**Figures 7A, B**). As the expression of the integrin αvβ8 gene in DCs was enhanced by the addition of *L. cremoris* YRC3780, we also analyzed the production of active TGF-β by adding latent TGF-β to the same culture system as above to investigate the possibility that *L. cremoris* YRC3780 would promote TGF-β activation in the coculture system. However, the amount of active TGF-β was below the detection limit (data not shown).

## 4 Discussion

Here, we used intestinal DCs to investigate the effects of lactic acid bacteria in a co-culture system of DCs and T cells, an *in vitro* experimental system that maximally mimics the intestinal immune system and in which antigen presentation occurs. We expect that this system reproduces the changes in immune responses that actually occur when lactic acid bacteria are orally ingested as a food component.

We found that *L. cremoris* YRC3780 upregulated the expression of several genes in MLN DCs that are involved in the induction of Tregs. We found increased gene expression of RALDH2 and integrin αvβ8, and for RALDH2, the percentage of DCs with actual enzyme activity increased. RALDH2 is an enzyme that converts retinal, derived from retinol, to retinoic acid, and retinoic acid promotes Treg induction (17). Integrin αvβ8 acts on latent TGF-β, an inactive form of TGF-β, to generate active TGF-β (18). Therefore, we consider that *L. cremoris* YRC3780 enhanced Treg induction by enhancing the production of retinoic acid and active TGF-β. Indeed, the addition of *L. cremoris* YRC3780 to the MLN DC and T cell coculture system increased the percentage of Foxp3^+^CD4^+^T cells. One study has shown that a *Lactobacillus* strain enhanced the RALDH activity of MLN DCs (3) and another study has reported that two probiotic *Lactobacillus* strains induced the expression of integrin αvβ8 (4). Nevertheless, our present study is a unique report demonstrating the enhancement of multiple Treg-inducing factors, including RALDH2 and integrin αvβ8, in intestinal DC by probiotic lactic acid bacteria, together with the induction of Foxp3^+^CD4^+^ T cells.

Because adding *L. cremoris* YRC3780 to MLN DC culture enhanced the gene expression of integrin αvβ8 which activates TGF-β, we tried to directly evaluate the activation of TGF-β by measuring active TGF-β, but the amount of active TGF-β in the culture supernatants did not change in MLN DC cultures and was below the detection limit in MLN DC and T cell cocultures. TGF-β binds to cells and various proteins such as latent TGF-β binding proteins (LTBPs), proteases and extracellular matrix proteins (19), and it is possible that this binding could have hindered its measurement.

We found an increase in the gene expression of IL-10 in MLN DC upon the addition of *L. cremoris* YRC3780. A high IL-10 environment promotes the differentiation of IL-10^+^Tregs (20). We observed an increase in the frequency of IL-10^+^Foxp3^+^CD4^+^T cells (**Figure 4C**), and it is possible that the increase in IL-10 production by DCs contributed to this increase. Although it was also possible that increase in IL-10 may have induced Foxp3^−^IL-10^+^T (Tr1) cells (21, 22), no significant increase in the percentage of these cells was observed (**Figure 4D**). Transcription factors associated with Tr1 cell development and function such as c-Maf and Egr-2 (22) were not examined in our microarray analysis. Although not strictly distinguishable, we consider that the effects of Tr1 cells were minimal because the frequency of Foxp3^+^IL-10^-^ cells was not changed significantly by the addition of strain YRC 3780.

Our microarray and subsequent qRT-PCR and ELISA analyses revealed that *L. cremoris* YRC3780 affected T cell differentiation. In the case of Th1-related genes, gene expression of *Tbx21* (T-bet), a master Th1 transcription factor (23), increased. This result indicated that differentiation into Th1 cells was promoted. In addition, gene expression of the Th1 cytokine IFN-γ was increased, and we confirmed that production of the protein was increased. With respect to the increased gene expression of IL-18R, the IL-18 receptor, and MyD88, a receptor adaptor molecule, it has been reported that Th1 cells are activated via the IL-18R/MyD88 signaling pathway (24). Therefore, our results may support the promotion of Th1 differentiation by *L. cremoris* YRC3780. With respect to Th2-related genes, the gene expression of GATA-3, a master transcription factor of Th2 cells (25), decreased. This result indicated that differentiation into Th2 was suppressed. The gene expression of IL-4 was also decreased, and we confirmed that production of the protein was reduced. Regarding the decrease in gene expression of the chemokine receptor CCR8, it has been reported that this chemokine receptor is highly expressed in Th2 cells and contributes to activation of the Th2 response (26, 27). Therefore, our results support the suppression of Th2 differentiation by *L. cremoris* YRC3780. In case of the Treg-related genes, gene expression of Foxp3, a master Treg transcription factor, tended to increase, and the percentage of Foxp3-expressing T cells actually increased in culture, indicating that differentiation into Tregs was promoted. In addition, the gene expression of furin, a proteolytic enzyme, increased. Furin may not be specifically expressed by Tregs, but it is known to generate TGF-β by acting on pro-TGF-β, the precursor of TGF-β containing the latency associated peptide and mature TGF-β, resulting in the induction of Tregs (19, 28, 29). Therefore, the increase in furin gene expression may support the promotion of Treg differentiation by *L. cremoris* YRC3780. In the case of Th17-related genes, although the gene expression of IL-17a increased, the signal intensity was very low. It was under the detection limit in seven out of eight samples used in the microarray analyses. In addition, the gene expression of RORγt, which is a master transcription factor of Th17 cells (30) was undetected. Furthermore, the expression of CCR6, a chemokine receptor known to be highly expressed in Th17 cells together with RORγt, decreased (30, 31). These results suggested that *L. cremoris* YRC3780 had only a limited impact on Th17 differentiation. The regulation of T cell subsets by *L. cremoris* YRC3780 seems to involve not only transcription factors and cytokines specific for T cell subsets, but also other related genes such as *Ccr5*, *Ccr6* and *Furin*.

Increased expression of *Aldh1a2*, *Itgav*, *Itgb8*, and *Il10* was observed in MLN DCs from TLR2-deficient mice treated with *L. cremoris* YRC3780, as well as in those from control mice. In addition, RALDH enzyme activity (i.e., the percentage of DCs with RALDH enzyme activity), was increased in both TLR2-deficient and control mice. This suggested that *L. cremoris* YRC3780 could stimulate MLN DCs through receptors other than TLR2 (**Figure 2**). Although we could not determine the pattern-recognition receptor(s) that recognized the component(s) of *L. cremoris* YRC3780, our results suggest that components of both the cytoplasm and the cell wall were recognized by the MLN DCs. The cytoplasmic fraction enhanced expression of the three of the genes examined: *Aldh1a2, Itgav*, and *Itgb8*. We further observed that the DNA and RNA fractions of *L. cremoris* YRC3780 increased the expression of *Itgb8* (**Supplementary Figure S4**). Although many lactic acid bacteria strains are recognized via TLR2 (2, 5, 16, 32, 33), there are also reports showing that DNA or RNA is the active component, being recognized by receptors such as TLR3 or TLR9 (2, 6, 16, 34, 35) This may also be the case for *L. cremoris* YRC3780 and different components of the cytoplasm might induce the expression of different genes. It was rather surprising that the cell-wall fraction of this gram-positive strain appeared to possess activity independently of TLR2. Perhaps pattern recognition receptors such as NOD-like receptors may be involved. A plasmacytoid DC-activating *Lactococcus lactis* subsp. *lactis* strain was reported to stimulate TLR4 (6), so the cell wall components may be recognized by a similar mechanism.

In this study, we used *L. cremoris* YRC3780 as the lactic acid bacterial strain. Our motivation for studying this strain was to elucidate the mechanism of its allergy-suppressing effect of this strain shown in clinical studies at the cellular level (11). In addition, although many studies have reported on the immunomodulatory functions of lactobacilli such as *Lactobacillus* (2, 16), there are few reports on lactococci such as *Lactococcus* (2, 6, 10). Our results suggest that induction of Tregs and inhibition of Th2 cell differentiation may be the mechanisms underlying the alleviation of allergies by this *Lactococcus* strain (11–13). Our results are consistent with the down-regulation of Th2 response observed by this strain in murine allergy models (12, 13). *L. cremoris* YRC3780 also enhanced the production of IL-10, an anti-inflammatory cytokine that is important for ameliorating inflammation, so this cytokine may also be involved in the antiallergic effect of *L. cremoris* YRC3780. In addition, another *L. cremoris* strain, JCM 16167 enhanced the expression of Treg-related genes in MLN DCs, suggesting that such activity may be observable in multiple *Lactococcus* strains. Furthermore, *Lactobacillus* strains have been reported to enhance ALDH activity, or the expression of integrin αvβ8 in intestinal DCs (3, 4). Therefore, it may be possible that other lactic acid bacteria, such as those in the *Lactobacillaceae* family can enhance both production of RALDH2 and TGF-β-activating integrins. These functions may have not been made clear in previous studies, because data on the gene-expression of MLN DCs are limited.

Since the intestinal immune response such as Treg generation is influenced by various factors, *in vitro* culture conditions may not completely mimic the internal intestinal environment. We are now setting up experiments to confirm our results in a pollen-allergy mouse model.

In summary, we analyzed the effects of *L. cremoris* strain YRC3780 on the immune responses induced by antigen presentation by intestinal DCs to T cells as well as mechanism of action of these immunomodulatory effects. The results suggest that *L. cremoris* YRC3780 promotes the induction of Th1 and Tregs (especially IL-10-hyperproducing Tregs) and regulated the Th1-Th2 and Treg-Th17 balances. Induction of Tregs may be unique to antigen presentation by intestinal DCs. Regulation of T-cell subsets involves many different genes. These properties may mediate the anti-allergic effects of lactic acid bacteria. In addition, the mechanism of action in DCs was suggested to be mediated by receptors other than TLR2. Our findings provides insights into the mechanism of the immunomodulatory effects of lactic acid bacteria on the intestinal immune system.

## Data availability statement

The data supporting this study are available from the corresponding author upon request.

## Ethics statement

The animal study was approved by The Experimental Animal Ethics Committee of the Graduate School of Agricultural and Life Sciences, the University of Tokyo (approval numbers P19-025, P21-044 and P22-056). The study was conducted in accordance with the local legislation and institutional requirements.

## Author contributions

RN: Conceptualization, Writing – original draft, Investigation. WG: Investigation, Writing – review & editing. HM: Investigation, Writing – review & editing. TT: Investigation, Writing – review & editing. SK: Investigation, Writing – review & editing. YW: Investigation, Writing – review & editing. IF: Resources, Writing – review & editing. KU: Methodology, Resources, Writing – review & editing. HNA: Writing – review & editing. SH: Conceptualization, Funding acquisition, Investigation, Project administration, Supervision, Writing – original draft, Writing – review & editing.
