# Supplementary material for: Lactococcus lactis subsp. cremoris YRC3780 modifies function of mesenteric lymph node dendritic cells to modulate the balance of T cell differentiation inducing regulatory T cells
Source: Front Immunol. 2024 Jul 8;15:1395380. doi: 10.3389/fimmu.2024.1395380 (PMC11261344; doi:10.3389/fimmu.2024.1395380)
Supplement: Supplementary file 1 [file DataSheet_1.pdf]

Supplementary Material

Supplementary Table

The sequences of the primers used are shown below.

Gapdh	
Forward	5’- TGT CCG TCG TGG ATC TGA C -3’
Reverse	5’- CCT GCT TCA CCA CCT TCT TG -3’
Aldh1a2	
Forward	5’- GAC TTG TAG CAG CTG TCT TCA CT -3’
Reverse	5’- TCA CCC ATT TCT CTC CCA TTT CC -3’
Itgav	
Forward	5’- GAG GGA GAT GTT CAC ACT TTG -3’
Reverse	5’- AGC AGG GAT TTC ACG TAC AG -3’
Itgb8	
Forward	5’- TGT ACT GAT CCC AGA AGC ATT G -3’
Reverse	5’- TGG GCC AGA AAA CAT TCT GAT -3’
Tgfb1	
Forward	5’- GGA CTC TCC ACC TGC AAG AC -3’
Reverse	5’- GAC TGG CGA GCC TTA GTT TG -3’
Il10	
Forward	5’- CCC AGA AAT CAA GGA GCA TTT G -3’

Reverse 5’- CAT GTA TGC TTC TAT GCA GTT G -3’

Il4

Forward 5’- GGT CTC AAC CCC CAG CTA GT -3’

Reverse 5’- GCC GAT GAT CTC TCT CAA GTG AT -3’

Ifng

Forward 5’- GCC ACG GCA CAG TCA TTG A -3’

Reverse 5’- TGC TGA TGG CCT GAT TGT CTT -3’

Foxp3

Forward 5’- CCC ATC CCC AGG AGT CTT G -3’

Reverse 5’- ACC ATG ACT AGG GGC ACT GTA -3’

Gata3

Forward 5’- GAA GGC ATC CAG ACC CGA AAC -3’

Reverse 5’- ACC CAT GGC GGT GAC CAT GC -3’

Tbx21

Forward 5’- CCA ACT GTC AAC TGC TTG G -3’

Reverse 5’- CCG CTT CAT AAC TGT GTT CC -3’

Il18r1

Forward 5’- CAC TGT CTT CTG CTG TCT GGA -3’

Reverse 5’- GAT GCA TGT TTA GGC TTC CAC -3

Ccr8

Forward	5’- GCA GTC TTT GAG GTG GAA GC -3’
Reverse	5’- GAT GGC TCT GGT CCT GTT GT -3’

Supplementary Figure S1

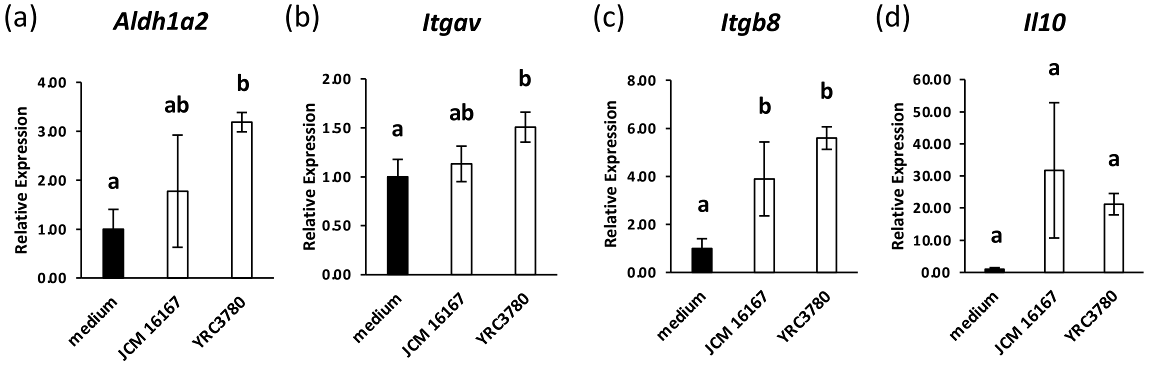

Supplementary Figure S1. The effect of *L. cremoris* JCM 16167 on the gene expression of Treg-inducing factors in MLN DC cultures.

MLN DC from BALB/c mice were cultured in the presence (10 μg/mL) of *L. cremoris* JCM 16167, *L. cremoris* YRC3780 or absence of bacterial preparation. Gene expression of *Aldh1a2* (a),*Itgav* (b), *Itgb8* (c) and *Il10* (d) was measured by qRT-PCR. Tukey's HSD test was used for statistical analysis. Values not sharing a common letter are significantly different.

Supplementary Figure S2

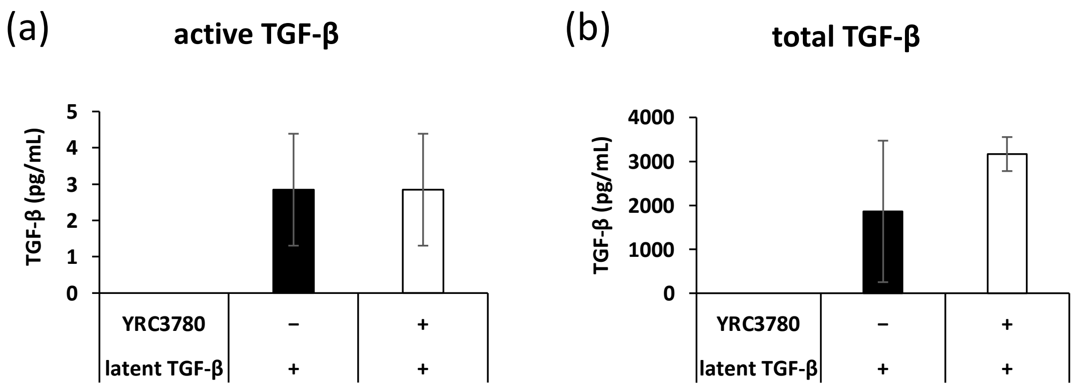

Supplementary Figure S2. Effects of *L. cremoris* YRC3780 on production of TGF-β in MLN DC cultures.

CD11c^{+}DCs from the MLNs of BALB/c mice were cultured in the presence (100 μg/mL) or absence of *L. cremoris* YRC3780 and latent TGF-β (10 ng/mL). The amounts of active (a) or total (b) TGF-β1 in the supernatants were analyzed by ELISA. Student's *t*-test was used for statistical analysis.

Supplementary Figure S3

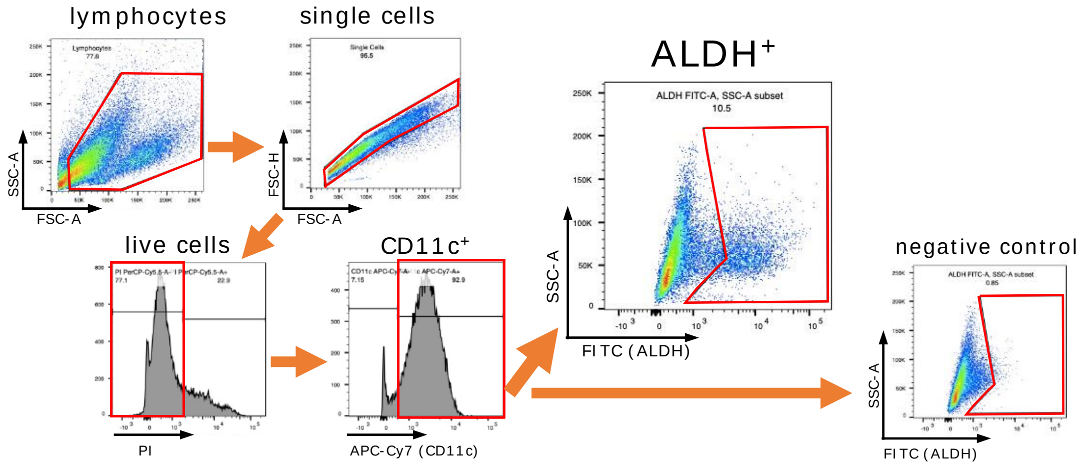

Supplementary Figure S3. Gating strategy used to identify ALDH^{+} cells among live CD11c^{+}DCs. CD11c^{+} cells were gated from single live lymphocytes and then ALDH^{+} cells were gated by measuring FITC. A negative control for ALDH^{+} was prepared by the addition of DEAB. The percentage of ALDH^{+}DCs was analyzed.

Supplementary Figure S4

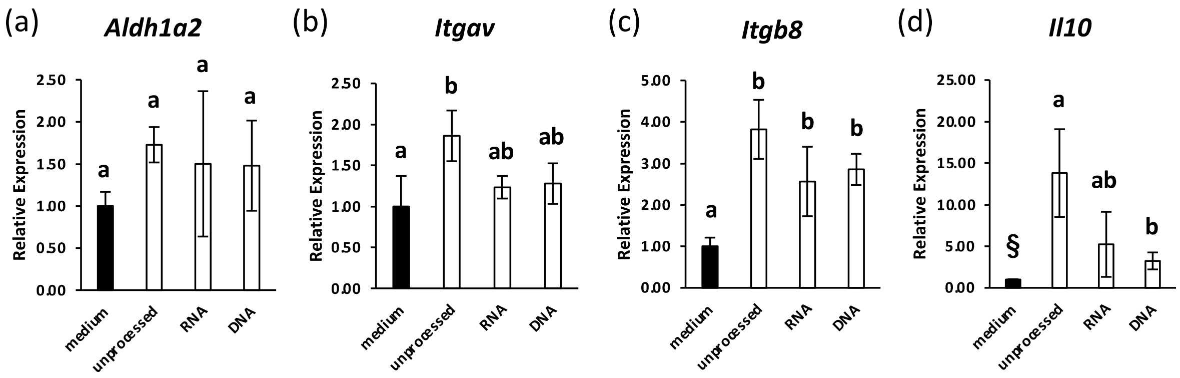

Supplementary Figure S4 Effects of cytoplasm, RNA and DNA fractions of *L. cremoris* YRC3780 on the gene expression of Treg-inducing factors in MLN DC cultures.

MLN DCs from BALB/c mice were cultured in the presence or absence of unprocessed *L. cremoris* YRC3780 or RNA or DNA fractions of *L. cremoris* YRC3780 (10 μg/mL) . Gene expression of *Aldh1a2* (a), *Itgav* (b), *Itgb8* (c) and *Il10* (d) was measured by qRT-PCR. Tukey's HSD test was used for statistical analysis. Values not sharing a common letter are significantly different. §: one of the three wells were not detected.

Supplementary Figure S5

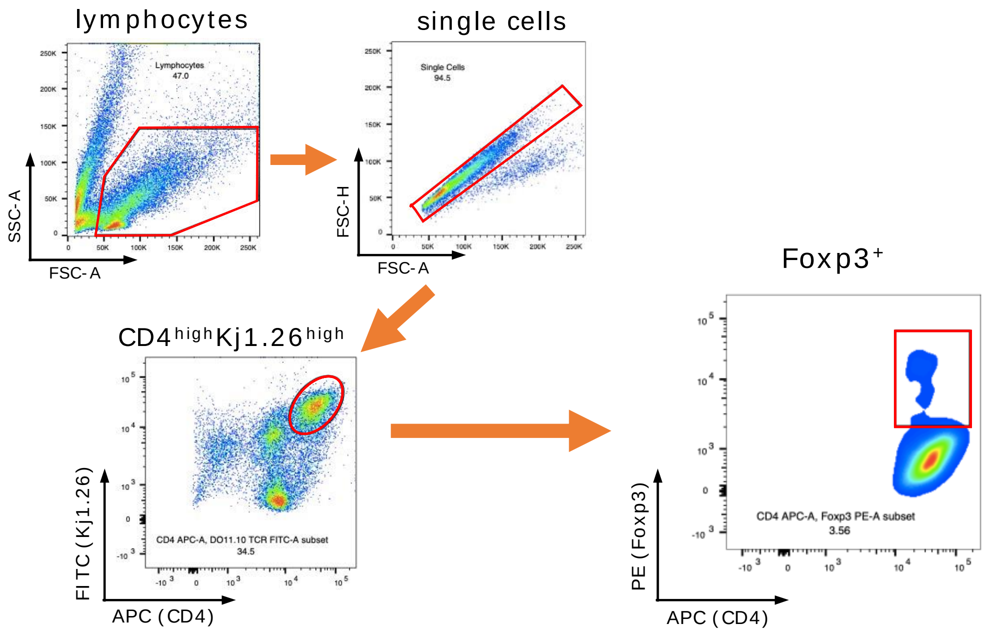

Supplementary Figure S5. Gating strategy used to identify Foxp3^{+}CD4^{+}T cells. CD4^{high}Kj1.26^{high} cells were gated from single lymphocytes and the percentage of Foxp3-expressing cells was analyzed.

Supplementary Figure S6

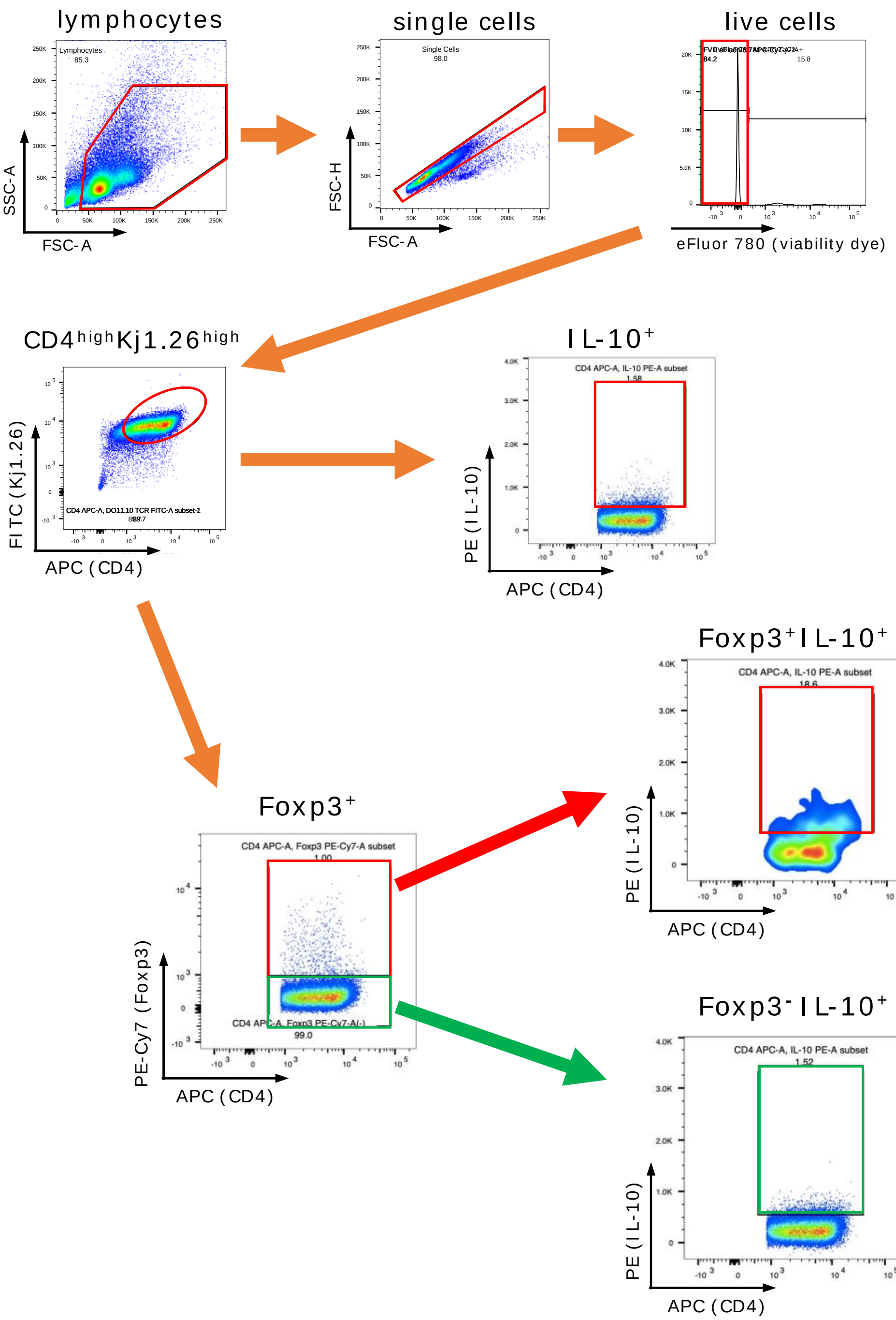

Supplementary Figure S6. Gating strategy used to identify IL-10^{+}, Foxp3^{+}IL-10^{+} and Foxp3^{-}IL-10^{+} CD4^{+}T cells.

CD4^{high}Kj1.26^{high} cells were gated from single live lymphocytes and the percentage of IL-10^{+}, Foxp3^{+}IL-10^{+} and Foxp3^{-}IL-10^{+} cells was analyzed.
